# The therapeutic potential of *Melissa officinalis* L. hydroalcoholic extract and rosmarinic acid in a rat asthmatic model: A study on anti-inflammatory and antioxidant effects

**DOI:** 10.22038/AJP.2023.23321

**Published:** 2024

**Authors:** Vahideh Abbasnia, Delaram Eslimi Esfahani, Mohammad Reza Khazdair, Shahrbanoo Oryan, Mohsen Foadoddini

**Affiliations:** 1 *Department of Animal Sciences, Faculty of Biological Sciences, Kharazmi University, Tehran, Iran*; 2 *Cardiovascular Diseases Research Center, Birjand University of Medical Sciences, Birjand, Iran*

**Keywords:** Melissa officinalis L., Antioxidant, Anti-inflammatory Experimental asthmatic model, Asthma

## Abstract

**Objective:**

The article studies how *Melissa officinalis* L. extract and rosmarinic acid (RA) affect lung inflammation, pathology, and oxidative stress in rats with ovalbumin-induced asthma.

**Materials and Methods:**

Asthma was induced in rats using ovalbumin injection and inhalation. The study assessed lung inflammation, pathological changes, and oxidative stress in control, untreated asthmatic rats and three treatment groups. These groups received *M. officinalis* extract (50, 100, 200 mg/kg), RA (0.5, 1, 2 mg/kg), or dexamethasone (Dex) 1 mg/kg.

**Results:**

In the sensitized group, white blood cell counts, malondialdehyde, and nitrite levels increased significantly, while thiol levels and the activity of superoxide dismutase and catalase decreased (p<0.001). However, all treatment groups with the extract, RA, and Dex showed a significant reduction in total white blood cells, eosinophils, monocytes, malondialdehyde, and nitrite levels compared to the asthma group (p<0.001 in all groups). Thiol levels and catalase and superoxide dismutase activity were significantly higher in all treated groups with RA and high extract doses (p<0.001). Lung pathological changes were also significantly less severe in the treated groups with dexamethasone, plant extract, and RA compared to the asthma group (p<0.05 to p<0.001).

**Conclusion:**

This study showed that *M. officinalis* and RA have antioxidant and anti-inflammatory effects in an animal asthma model, suggesting their potential for treating asthma symptoms.

## Introduction

Asthma is a chronic inflammatory disease characterized by coughing, wheezing, and difficulty breathing. Recent statistics indicate that worldwide, 300 million people are affected by this condition, with 1,000 people dying daily (Agache and Akdis, 2019; Shariat et al., 2020). Both developed and developing countries are witnessing an increasing prevalence of this disease (Varmaghani, 2016). Multiple factors, including environmental, genetic, and epigenetic elements, contribute to asthma development and its response to treatment (Cevhertas, 2020b). Inflammation dominates the pathophysiology of asthma, with the activation of mast cells and damage to epithelial cells leading to the release of inflammatory and immunological mediators in the airways. Additionally, inflammatory cells like eosinophils, lymphocytes, neutrophils, and monocytes contribute to airway inflammation (Bateman et al., 2008; Salehi et al., 2011a).

Inflammation and oxidative stress are interconnected, as an increase in one leads to an increase in the other. Healthy lungs possess high antioxidant resources to protect against oxidative damage (Comhair et al., 2000). However, individuals with asthma show an imbalance between oxidant and antioxidant compounds, as evidenced by the results. Asthma patients exhibit high levels of malondialdehyde and low levels of antioxidants such as superoxide dismutase, thiol groups, and catalase. These findings suggest a significant role for these factors in the pathophysiology of asthma (Eftekhar et al., 2018).

Even though inhaled corticosteroids (ICS) reduce the risk of asthma symptoms and mortality (Borna et al., 2019; Papi et al., 2018), current asthma treatments primarily focus on relieving symptoms. Targeting natural anti-asthma drugs with minimal side effects aims to improve asthma symptom control in severe asthma patients and mitigate the side effects of oral corticosteroids (OCS). Evidence suggests Medicinal plants can yield effects akin to dexamethasone in treating asthma (Eftekhar et al., 2019). Within the Lamiaceae family, one can find *Melissa officinalis* (*M. officinalis*) a well-established plant with a rich history in traditional medicine for addressing many ailments. Over two millennia have been dedicated to its utilization, and numerous studies have sought to unveil its medicinal attributes. This botanical gem has been employed for centuries to alleviate maladies such as headaches, digestive disorders, nervous afflictions, and rheumatoid arthritis (Capecka and Mareczek, 2005; Jun et al., 2012). Notably, the hepatoprotective effects of *M. officinalis* alcoholic extract have manifested through the reduction of liver enzyme levels of cholesterol and an enhancement in liver function (Zarei et al., 2014). *M. officinalis* flourishes as a perennial herbaceous plant in central and southern Europe, Iran, and Central Asia, and its versatile nature has led to global cultivation (Behnam Rassouli et al., 2010).

Several studies have meticulously examined the anti-inflammatory properties inherent in the leaves of *M. officinalis*. In the annals of traditional medicine, essential oils have been a stalwart in treating various inflammatory and painful conditions, owing to their well-documented anti-inflammatory attributes (Bounihiet al., 2013). The amalgamation of rosmarinic acid (RA), terpenoids, and flavonoids within *M. officinalis* extract drives its anti-inflammatory and analgesic prowess. Within the plant, RA and flavonoids effectively impede inflammatory enzymes such as cyclooxygenase, lipoxygenase, and monooxygenase (Miladi Gorgi et al., 2005; Petersen and Simmonds, 2003).

Researchers have conducted comprehensive investigations into the effects of *M. officinalis* extract on skin cells containing RA, both in regular and oxidative stress conditions. Studies involving human keratinocyte cells have demonstrated a remarkable 28% reduction in intracellular ROS and the fortification of cells against oxidative damage induced by H2O2 (Ramanauskienė et al., 2015).

In the realm of phenolic compounds, *M. officinalis* boasts RA as the most abundant and gallic acid as the least (Arceusz and Wesolowski, 2013). Researchers have underscored the significance of RA within *M. officinalis* as a pivotal phenolic compound (Toth et al., 2003). Renowned for its potent antioxidant properties, this phenolic compound has been shown to mitigate inflammation and oxidative stress. It has been convincingly demonstrated that RA inhibits various inflammatory diseases, including arthritis, colitis, and atopic dermatitis, both in vitro and in vivo (Chunxu and Lin, 2020).

The current study investigated the potential protective effects of the hydroalcoholic extract of *M. officinalis* and its active ingredient, RA, against ovalbumin-induced bronchial asthma in rats. Specifically, this research focused on evaluating the impact of these natural compounds on the total white blood cell count and various types of white blood cells serving as markers of inflammation. Furthermore, it was designed to assess the levels of oxidative stress factors in serum, including malondialdehyde (MDA), catalase (CAT), and superoxide dismutase (SOD). Additionally, pathological alterations in the lung were assessed to gauge the effectiveness of these natural compounds in alleviating asthma symptoms.

By examining these pivotal indicators, this study aims to contribute to the advancement of natural and efficacious treatments for asthma, thereby reducing dependence on conventional drugs with potential adverse side effects. Since asthma is a significant public health concern, identifying alternative, natural, and effective therapies is paramount in enhancing the quality of life for individuals affected by this condition.

## Materials and Methods

**Plant extracts preparation**

For this study, we obtained the *M. officinalis* plant from Tabas City, situated in South Khorasan province. A botanical expert from the Birjand Center of Payam Noor University confirmed the plant's identification (voucher number: L-110). We employed 300 g of powdered leaves from the plant to prepare the hydroalcoholic extract. Initially, the powdered leaves were dissolved in a solution comprising 80% methanol (CH₃OH) and 20% water (H₂O).

In the subsequent step, the hydroalcoholic solution containing the sample was filtered through filter paper after 24 hours. Once the methanol had been eliminated from the solution, we subjected it to evaporation using a Heidolph rotary evaporator. This process was continued until a dry extract was achieved (Ardei, 2015).

The hydroalcoholic extracts of the *M. officinalis* plant were subjected to testing by the Iranian Forestry and Pasture Research Institute using high-performance liquid chromatography (HPLC) to identify Rosmarinic Acid (RA) as the active ingredient. Additionally, a ready-to-use form of RA was procured from Sigma Co., Germany.Top of Form

**Animals grouping**

The male Wistar rats were categorized into nine groups, with six rats in each group, as outlined below: I) Non-asthmatic control group. II) Asthmatic group. III) Asthmatic group treated with dexamethasone at a dosage of 1 mg/kg/day via oral gavage for 21 days. IV-VI) Asthmatic groups were exposed to three concentrations (200 mg/kg/day, 50 mg/kg/day, and 100 mg/kg/day) of *M. officinalis* hydroalcoholic extract through oral gavage for 21 days. VII-IX) Asthmatic groups were administered with three different concentrations of RA (0.5 mg/kg/day, 1 mg/kg/day, and 2 mg/kg/day) via oral gavage for a 21-day duration (Dehbani Z., 2019).

**Study protocol**

To sensitize animals and induce experimental asthma in rats, the following protocols were adhered to:

To develop experimental asthma, which is more accurately referred to as sensitization, male Wistar rats received injections and inhalations of ovalbumin (98% pure, Sigma, St. Louis, Missouri, USA) over 21 days. In the experiment, 1 mg/kg of ovalbumin was intraperitoneally injected along with 100 mg of aluminum hydroxide on the first, second, and third days. Subsequently, the animals were exposed to 1% ovalbumin inhalation for 20 minutes on days 6, 9, 12, 15, 18, and 21 (Kianmeher et al., 2016).

**Blood sampling and serum isolation**

The rats were anesthetized intraperitoneally at the end of the experiment with 10% ketamine (80 mg/kg) and 2% xylazine (10 mg/kg). Blood samples were collected directly from the heart of each animal after anesthesia. To determine WBC types and counts, 2 ml of blood was placed in a test tube containing ethylenediaminetetraacetic acid (EDTA). The blood sample was then centrifuged at 3500 rpm for 10 minutes. Finally, the supernatants from this reaction were collected, stored at -80°C, and subsequently measured for malondialdehyde (MDA), superoxide dismutase (SOD), and catalase (CAT).

**Determination of total and differential WBC cell counts**

We determined the total number of WBCs using the subtractive counting method, widely employed for determining WBC counts. Furthermore, the types of WBCs and their respective percentages were determined by spreading the blood samples on slides and staining them with Wright-Giemsa. This staining technique can distinguish and identify different types of WBC based on their morphological characteristics.

**Determination of oxidative stress parameters**

We measured the levels of MDA (Janero, 1990), and the activities of SOD (Madesh and Balasubramanian, 1998) and CAT (Aebi, 1984) enzymes, as well as nitrite (Yousefniapasha et al. 2015) levels, using assay kits provided by Carmania Parsgen Company, Iran. Additionally, the total content of thiol groups was determined using an assay kit from Koush Aria Azma, Iran, following the manufacturer's instructions.

**Histological examination**

The lungs were dissected and immersed in a 10% formalin solution overnight. Following dehydration, the samples were encased in paraffin, and block sectioning (5 µm) was carried out using a microtome. Subsequently, the sections were subjected to hematoxylin-eosin (H&E) staining and examined under a light microscope (Eftekhar et al., 2019).

**Ethical considerations**

The experiment involved male Wistar rats with an approximate weight range of 20±0.2 g. These rats were sourced from the Animal Breeding Center at Birjand University of Medical Sciences. The animal housing facility's environmental conditions were meticulously maintained at 22±2°C, with a 12-hour light-dark cycle and a 50-60% humidity level. It is essential to note that all laboratory procedures conducted in this study received ethical approval from the Birjand University of Medical Sciences Ethics Committee under protocol number IR.BUMS.REC.1400.272.

**Statistical analysis**

All data were reported as mean±SEM. To compare data across the treated groups, the untreated asthma group, and the control group, we employed a one-way analysis of variance (ANOVA), followed by the Tukey-Kramer posttest analysis using the SPSS software. Statistical significance was considered when p<0.05.

## Results

**The Impact of** ***M. officinalis*** **extract and RA on WBC count and types**

**Figure 1:**
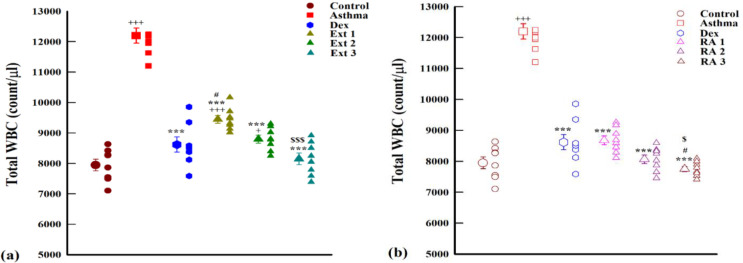
The mean±SEM of total WBC count (count/ml) in the blood of non-treated sensitized animals (asthmatic model) and treated with dexametason (Dex) and *M. officinalis* extracts (50, 150 and 200 mg/kg), Ext 1, Ext 2 and Ext 3, respectively) (a) and rosmarinic acid (0.5, 1 and 2 mg/kg) (RA 1, 2 and 3, respectively) (b), (n=8, for all groups). Comparison of the data between different groups were done using one-way analysis of variance (ANOVA) with Tukey-Kramer posttest. +: p<0.05 and +++: p<0.001, compared between control and other groups. ***: p<0.001, compared between Asthma and other groups. #: p<0.05, compared between Dex and other treated groups. $: p<0.05 and $$$: p<0.001, compared between extract Ext 50 vs Ext 100 and 200 and RA 0.5 vs RA 1 and 2.

A notable increase in WBC count was observed in the induced asthma group compared to the control group (p<0.001). The total WBC count exhibited a significant reduction in all treatment groups that received extracts at doses of 50, 100, and 200 mg/kg, as well as in all treatment groups administered RA at 0.5, 1, and 2 mg/kg, along with the dexamethasone group (p<0.001). Moreover, at high concentrations, RA exhibited a significant decrease compared to dexamethasone (p<0.05). Additionally, treatment with higher doses of extracts (200 mg/kg) and RA (2 mg/kg) showed significant improvement when compared to the lower doses of extract and RA (50 and 0.5 mg/kg, respectively) (Figure 1a and b).

The asthmatic group displayed elevated levels of neutrophils, eosinophils, and monocytes compared to the control group (p<0.001). Conversely, the asthmatic group exhibited a significantly lower percentage of lymphocytes than the control group (p<0.001) (Tables 1 and 2).

It was noted that lymphocyte percentages were significantly higher in all treatment groups using RA (0.5, 1, and 2 mg/kg), as well as in the high-dose extract groups (200 and 100 mg/kg) and the dexamethasone group when compared to the asthmatic group (p<0.001). The percentage of lymphocytes increased significantly in the high-dose treatment groups of the extract (200 and 100 mg/kg) and RA (2 mg/kg) compared to the low-dose treatment groups (50 and 0.5 mg/kg, respectively) (p<0.001). While the percentage of lymphocytes in the high-concentration extract group (200 mg/kg) was not significantly different from the dexamethasone treatment group, it was higher in the high-concentration treatment group (2 mg/kg) when compared with the dexamethasone group (p<0.001) and the control group (Table 1 and 2).

**Table 1:** Differential WBC count in the blood of different treated groups

		**Control**	**Asthma**	**Dex**	**Ext ** **50 mg/kg**	**Ext ** **100 mg/kg**	**Ext ** **200 mg/kg**
**Lymphocyte**	74.28±1.64		62.57±1.46 ^+++^	70.58±1.55 ^+++***^	62.13±1.19 ^+++###^	68.001±1.18 ^+++***##$$$^	69.86±1.41 ^+++***$$$^
**Neutrophil**	20.60±1.08		28.30±1.02 ^+++^	23.60±1.36 ^+++***^	26.65±1.54 ^+++###^	25.57±0.94 ^+++***#^	23.89±1.36 ^+++***$$$^
**Eosinophil**	2.19±0.28		4.08±0.63 ^+++^	2.60±0.39 ^***^	6.23±0.56 ^+++***###^	3.01±0.33 ^+***$$$^	2.90±0.50 ^+***$$$^
**Monocyte**	2.92±0.53		5.03±0.80 ^+++^	3.21±0.48 ^***^	4.98±0.49 ^+++###^	3.40±0.46 ^***$$$^	3.34±0.25 ^***$$$^

The meanSEM values of lymphocytes, neutrophils, eosinophils and monocytes percentages in the blood of control, non-treated sensitized animals (asthmatic model) and treated with dexametason (Dex) and *M. officinalis* extracts (Ext), (n=8, for all groups). The comparisons of the data between groups was done using One way analysis of variances (ANOVA) with Tukey Kramer post test.+: p<0.05 and +++: p<0.001, compared between control and other groups. ***: p<0.001, compared between Asthma and other groups. ###: p<0.001, compared between Dex and other treated groups. $$$: p<0.001, compared between Ext 50 vs Ext 100 and 200.

**Table 2:** Total and differential WBC count in the blood of in different treated groups

	**Control**		**Asthma**	**Dex**	**RA ** **0.5 mg/kg**	**RA ** **1 mg/kg**	**RA ** **2 mg/kg**
**Lymphocyte**		74.28±1.64	62.57±1.46 ^+++^	70.58. ±1.55 ^+++***^	71.19±1.12 ^++***^	71.87±1.50 ^+***^	76±1.40 ^***###$$$^
**Neutrophil**		20.60±1.08	28.30±1.02 ^+++^	23.60±1.36 ^+++***^	22.68±0.99 ^+***^	22.39±1.50 ^***^	21.02±1.26 ^***##^
**Eosinophil**		2.19±0.28	4.08±0.63 ^+++^	2.60±0.39 ^***^	3.17±0.60 ^++***^	3.00±0.46 ^+***^	1.86±0.23 ^***#$$$^
**Monocyte**		2.92±0.53	5.03±0.80 ^+++^	3.21±0.48 ^***^	2.95±0.54 ^***^	2.73±0.29 ^***^	1.11±0.14 ^+++***###$$$^

The meanSEM values of lymphocytes, neutrophils, eosinophils and monocytes percentages in the blood of control, non-treated sensitized animals (asthmatic model) and treated with dexametason (Dex) and rosmarinic acid (RA), (n=8, for all groups). The comparisons of the data between groups was done using One way analysis of variances (ANOVA) with Tukey Kramer post test. +: p<0.05, ++: p<0.01 and +++: p<0.001, compared between control and other groups. ***: p<0.001, compared between Asthma and other groups. #: p<0.05, ##: p<0.01and ###: p<0.001, compared between Dex and other treated groups. $$$: p<0.001, compared between RA 0.5 vs RA 1 and 2 mg/kg.

In comparison to the asthmatic group, the percentage of neutrophils and monocytes significantly decreased (p<0.001) in the dexamethasone group and the high-dose extract treatment groups (200 and 100 mg/kg), as well as in all three treatment groups containing RA (0.5, 1, and 2 mg/kg). Furthermore, compared to the dexamethasone group, the percentage of neutrophils decreased significantly in the high-dose RA treatment group (2 mg/kg). The percentage of neutrophils significantly decreased in the high-dose extract treatment group (200 mg/kg) when compared to the low-dose extract treatment group (50 mg/kg) (p<0.001). Dexamethasone and extract treatment groups (50, 100, and 200 mg/kg), as well as RA treatment groups (0.5, 1, and 2 mg/kg), exhibited significantly lower levels of eosinophils than the asthma group (p<0.001). A noteworthy decrease in eosinophils and monocytes was observed as the dose increased. Consequently, treatment with higher doses of extracts (100 and 200 mg/kg) and RA (2 mg/kg) demonstrated a significant decrease compared to the lower doses of extract and RA (50 and 0.5 mg/kg) (p < 0.001 and p<0.05, respectively) (Table 1 and 2).

**Effects of** ***M. officinalis*** **extract and RA on oxidant and antioxidant factors**

**Figure 2:**
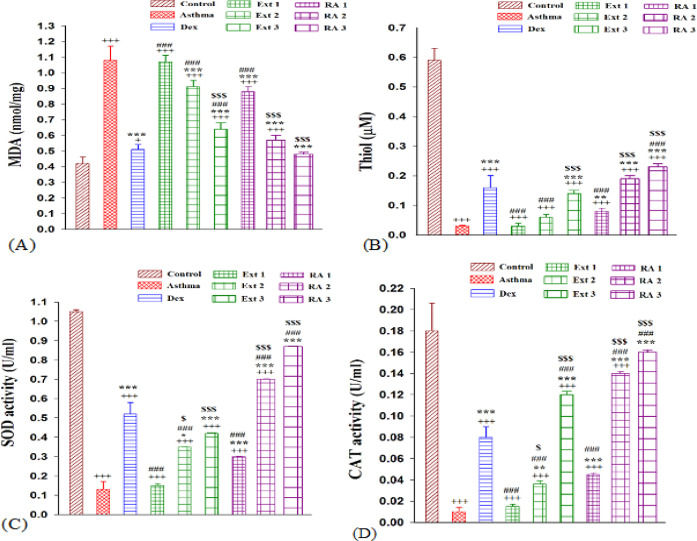
The mean±SEM level of MDA (A), Thiol (B), activities of SOD (C) and CAT(D) in the serum of non-treated sensitized animals (asthmatic model) and treated with dexametason (Dex) and *M. officinalis* extracts (50, 150 and 200 mg/kg), Ext 1, Ext 2 and Ext 3, respectively) and rosmarinic acid (0.5, 1 and 2 mg/kg) (RA 1, 2 and 3, respectively), (n=8, for all groups). Comparison of the data between different groups were done using one way analysis of variance (ANOVA) with Tukey-Kramer posttest. +: p<0.05 and +++: p<0.001, compared between control and other groups. ***: p<0.001, compared between Asthma and other groups. #: p<0.05 and ###: p<0.001, compared between Dex and other treated groups. $: p<0.05 and $$$: p<0.001, compared between extract Ext 50 vs Ext 100 and 200 and RA 0.5 vs RA 1 and 2.

Asthmatic groups exhibited a significant increase in MDA levels compared to the control group (p<0.001). MDA levels were lower in all treatment groups receiving the extract, Ros A, and dexamethasone, except for those receiving a low dose of the plant extract (50 mg/kg) when compared to those receiving the asthma medication (p<0.001). The MDA level was not significantly different from the control group at the high concentration of RA (2 mg/kg). It was determined that all three treatment groups receiving the extract (100 mg/kg, 200 mg/kg, and 50 mg/kg) and those receiving a low dose of RA (0.5 mg/kg) had significantly higher levels of MDA than the dexamethasone group (p<0.001) (Figure 2A).

The thiol level significantly decreased in sensitized groups compared to the control group (p<0.001). All treatment groups, including dexamethasone, all doses of RA (0.5 mg/kg, 1 mg/kg, and 2 mg/kg), and the higher dose of extract (200 mg/kg), demonstrated a significant increase in the levels of thiol (p<0.01 to p<0.001). Thiol levels significantly increased in the higher plant extract as well as the medium and higher doses of RA treatment groups (100 mg/kg and 200 mg/kg) compared to the low-dose extract and RA treatment group (p<0.001 for all cases) (Figure 2B).

There was a significant difference in SOD and CAT activity levels between the asthmatic and control groups (p<0.001 for both cases). All treatment groups, including dexamethasone, RA (0.5 mg/kg, 1 mg/kg, and 2 mg/kg), and extract (100 mg/kg and 200 mg/kg), showed a significant increase in levels of SOD and CAT activities (p<0.01 to p<0.001). Compared to the dexamethasone group, SOD activity was significantly lower in the plant extract (50 mg/kg, 100 mg/kg, and 200 mg/kg) and the low-dose RA treatment groups. However, the medium and higher doses of the RA treatment group (1 mg/kg and 2 mg/kg) demonstrated a significant increase in levels of SOD and CAT activity (p<0.001 for both cases) (Figure 2 C and D).

**Figure 3:**
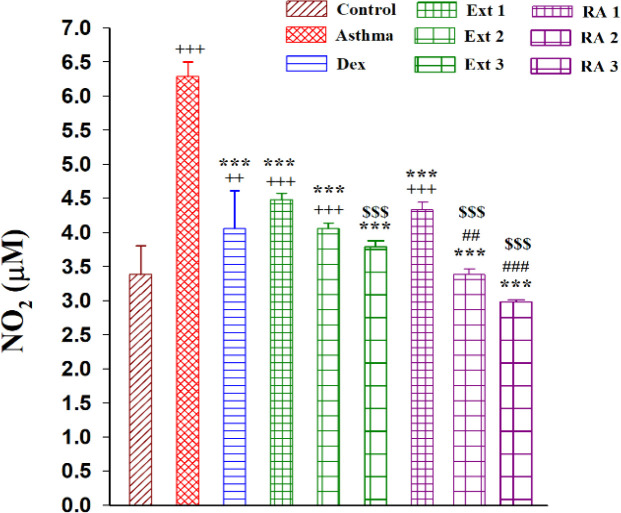
The mean±SEM level of No_2_ in the serum of non-treated sensitized animals (asthmatic model) and treated with dexametason (Dex) and *M. officinalis* extracts (50, 150 and 200 mg/kg), Ext 1, Ext 2 and Ext 3, respectively, and rosmarinic acid (0.5, 1 and 2 mg/kg) (RA 1, 2 and 3, respectively) (b), (n=8, for all groups). Comparison of the data between different groups were done using one way analysis of variance (ANOVA) with Tukey-Kramer posttest. ++: p<0.01 and +++: p<0.001, compared between control and other groups. ***: p<0.001, compared between Asthma and other groups. ##: p<0.01 and ###: p<0.001, compared between Dex and other treated groups. $$$: p<0.001, compared between extract Ext 50 vs Ext 100 and 200 and RA 0.5 vs RA 1 and 2.

The level of NO_2_ also significantly increased in the asthma group compared to the control group (p<0.001). Treatment with Dex, plant extracts, and RA significantly reduced the level compared to the asthma group (p<0.001). NO_2 _levels significantly decreased in the medium and higher concentrations of RA (1 mg/kg and 2 mg/kg) compared to the Dex treatment group (p<0.001 for both cases). Furthermore, treatment groups receiving the higher dose of extract (200 mg/kg) and medium and higher concentrations of RA (1 mg/kg and 2 mg/kg) showed a significant increase in NO_2_ levels compared to the lower dose of extract and RA (p<0.001 for all cases) (Figure 3). 

**Effects of** ***M. officinalis*** **extract and RA on histological changes**

The lung pathology observed in the control and sensitized groups included inflammation, muscle hypertrophy, and mucus production. An evaluation of the pathological changes indicated that all sensitized groups induced by ovalbumin exhibited significantly more pronounced pathological changes than the control group. Treatment with dexamethasone and *M. officinalis* extracts, particularly at higher doses of 100 and 200 mg/kg, effectively ameliorated these changes. Sensitized groups that received no treatment and those treated with a lower dose of the extract (50 mg/kg) displayed significantly more pronounced pathological changes in terms of inflammation, muscle hypertrophy, and mucus plaques when contrasted with the control group (p<0.001 and p<0.05, respectively). The treatment with dexamethasone and *M. officinalis* extract at doses of 50, 100, and 200 mg/kg significantly decreased these pathological changes in a dose-dependent manner (p<0.05 to p<0.001). Furthermore, the higher dose of the extract (200 mg/kg) significantly reduced inflammation in comparison to the dexamethasone treatment group (p<0.01) (Table 3).

**Figure 4:**
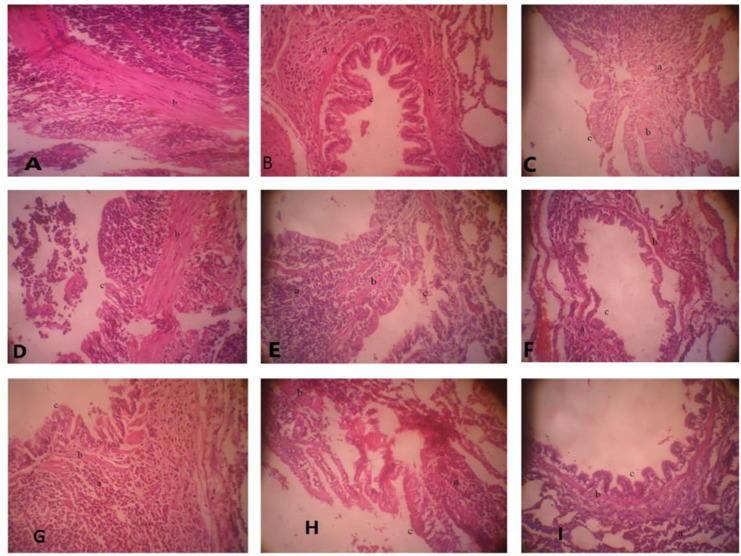
The lung specimens in asthma (A), control (B) and non-treated sensitized animals (asthmatic model) and treated with dexametason (C) and *M. officinalis* extracts (50, 150 and 200 mg/kg), (D, E and F, respectively) and rosmarinic acid (RA 1, 2 and 3), ), (G, H and I, respectively) Lung inflammation (a), muscle hypertrophy (b) and Mucine (c), (magnification 10 x 40).

Both the control and sensitized groups displayed signs of lung inflammation and hypertrophy. However, the sensitized group exhibited more severe pathological changes than the control group, as indicated by scoring these changes. Dexamethasone and RA effectively mitigate these pathological changes, especially at higher dosages (1 and 2 mg/kg). The sensitized groups that did not receive any treatment exhibited significantly higher levels of inflammation, muscle hypertrophy, and mucus plaques than the control groups. The inflammation index and the response to lower doses of RA (0.5 mg/kg) were notably higher in the RA treatment group compared to the control group. Treatment of the asthma group with dexamethasone and higher doses of RA (1 and 2 mg/kg) resulted in significantly lower blood pressure than treatment with dexamethasone alone. Additionally, sensitized animals treated with dexamethasone and RA at various dosages (0.5, 1, and 2 mg/kg) significantly reduced muscle hypertrophy in a dose-dependent manner compared to the asthma group. Furthermore, sensitized animals treated with dexamethasone and RA exhibited significantly fewer mucus plaques than asthmatic animals (see Figure 4 and Table 3).

**Table 3:** The pathological changes in the studied groups

**Parameters**	**Control**	**Asthma**	**Dex**	**Ext 50 mg/kg**	**Ext 100 mg/kg**	**Ext 200 mg/kg**	**RA 0.5 mg/kg**	**RA 1 mg/kg**	**RA 2 mg/kg**
Lung inflammation	0.37±0.41	2.5±0.5 ^+++^	0.75±0.43 ^***^	1.75±0.43 ^+^	1±0.35 ^**^	0.25±0.43 ^***$$^	1.75±0.43 ^++^	1.25±0.43 ^*^	0.75±0.25 ^***^
Muscle hypertrophy	0.5±0.35	2.62±0.41 ^+++^	1±0.35 ^***^	1.25±0.43 ^**^	1±0.35 ^***^	0.87±0.21 ^***^	1.37±0.41 ^**^	1.12±0.21 ^**^	0.62±0.41 ^***^
Mucus plaques	0.25±0.43	2.25±0.43^ +++^	0.62±0.41 ^**^	1.34±0.41 ^+^	1.12±0.21 ^*^	0.87±0.21 ^**^	1.12±0.21 ^*^	0.87±0.21^**^	0.75±0.55 ^**^

The meanSEM values of the pathological changes including; lung inflammation (a), muscle hypertrophy (b) and mucus plaques (c), in control, non-treated sensitized animals (asthmatic model) and treated with dexametason (Dex), *M. officinalis* extracts (Ext) 50, 150 and 200 mg/kg, and rosmarinic acid (RA). Comparison of the data between groups was done using one way analysis of variance (ANOVA) with Tukey-Kramer posttest.+; p<0.05, ++: p<0.01 and +++: p<0.001, compared between control and other groups. *: p<0.05, **: p<0.01 and ***: p<0.001, compared between Asthma and other groups. $$: p<0.01, compared between Ext 50 and 200.

## Discussion

Asthma is a chronic inflammatory disease that afflicts millions of people worldwide. This condition results from persistent airway inflammation, leading to alterations in the structure and function of the respiratory passages, non-specific hyper-responsiveness of the bronchi, and airway obstruction (Nakagome and Nagata, 2011). A connection exists between airway inflammation and releasing immune cells and other mediators (Salehi et al., 2011b). Numerous studies have illustrated that reactive oxygen species (ROS) trigger and intensify inflammatory responses in individuals with asthma who experience recurrent episodes of airway obstruction (Nadeem et al., 2008). Hence, anti-inflammatory and antioxidant compounds are currently employed to manage this condition effectively. *M. officinalis* is a medicinal herb renowned for its multifaceted therapeutic properties (Capecka and Mareczek, 2005; Jun et al., 2012). Flavonoids have been derived from *M. officinalis* extracts, and phenolic compounds such as Ros A have been associated with the plant's antioxidant and anti-inflammatory effects (A Seif El-Dein et al., 2023; Safaeian et al., 2016).

In the context of this study, we examined the impact of *M. officinalis* hydroalcoholic extract and RA - its active ingredient - on rats to assess their effects on lung tissue pathology, total white blood cell count, white blood cell type composition, and levels of oxidants (MDA and No_2_) and antioxidants (thiol, CAT, SOD) in the serum. Recent research has revealed that the levels of oxidative stress in the plasma of asthma patients can serve as practical indicators of disease severity (Ammar, 2022). By prior investigations, asthma patients who did not receive treatment exhibited an increase in total white blood cells, neutrophils, eosinophils, and monocytes, along with a decrease in lymphocytes in their serum. This observation aligns with earlier studies (Boskabady, 2010; Shakeri et al., 2017) and substantiates the induction of asthma in the animal model. Eosinophils and neutrophils, which increase in number in asthmatic patients, play a pivotal role in asthma pathogenesis. Asthma is a pathogenic condition driven by an elevated count of white blood cells like eosinophils and neutrophils. These processes can produce many products, including cytokines, thromboxane, metalloproteinases, reactive oxygen species, and NO, all of which can impact the airway's structure, mucus production, and responses (Bloemen et al., 2007).

In the treatment groups that received the hydroalcoholic extract of the plant and those that received Ros A, there was a notable decrease in the total white blood cell count and the percentages of eosinophils, monocytes, neutrophils, and lymphocytes. Additionally, there was an increase in the percentage of lymphocytes in the treatment groups that received RA. A previous study had demonstrated that an animal model of asthma could benefit from a hydroalcoholic extract of saffron, resulting in reduced total and differential white blood cells (WBC), similar to the protective effects observed with *M. officinalis* and Ros A in rats with asthma induced by ovalbumin (Vosooghi et al., 2013). The current research suggests that these changes may be attributed to the anti-inflammatory effects of this plant and its active ingredient.

Several studies have demonstrated the positive effects of *M. officinalis* on the immune system response in mice (Drozd and Anuszewska, 2003). One study reported that flavonoids in the plant inhibit the cyclooxygenase enzyme, which, in turn, inhibits the production of prostaglandins and inflammatory cytokines during inflammation (Vaez et al., 2011). Furthermore, research has shown that RA improves colitis caused by dextran sulfate sodium (DSS). It was found that RA reduces inflammation and the production of interleukin-1 beta, interleukin-22, and interleukin-6 and inhibits COX2 production (Jiang et al., 2018) in that research.

*Ocimum gratissimum* and RA were also investigated in another study for their effects on asthma and immune function. It was found that RA significantly increased neutrophils and decreased eosinophils in rats induced with the mite Blomia tropicalis in the study (Costa et al., 2012). These findings are consistent with the results of the mentioned studies, indicating that *M. officinalis* and RA possess anti-inflammatory properties.

Antioxidants present in the lungs prevent tissue damage caused by inhaling external or endogenous oxidants (Kirkham and Rahman, 2006). However, asthma-induced inflammation disrupts the balance of oxidant and antioxidant factors (Roshanzamir and Vahdat, 2011; M. Sahiner et al., 2018). As a result of increased free radical production and oxidative stress, ovalbumin has been shown to facilitate asthma disease models in animals (Aono et al., 2014; Yousra M. Ezz-Eldina, 2020). According to data analysis, OVA causes severe oxidative stress, exacerbated by decreased SOD and CAT activity and increased MDA levels in rats sensitized with ovalbumin. These findings are consistent with previous research (M. Ezz-Eldina et al., 2020).

An asthma attack increases malondialdehyde, a lipid peroxidation product (Ammar, 2022). The SOD enzyme is a metalloprotein that initiates the first step in antioxidant defense, reducing ROS to form two less harmful oxidative compounds, namely hydrogen peroxide and oxygen (Aldini et al., 2011; Gomes et al., 2012). Consequently, superoxide radicals cannot damage tissues this way (Zinellu et al., 2016). Asthmatic patients exhibit altered SOD activity (Brown Las, 2007). Catalase, the most abundant antioxidant enzyme containing iron, converts hydrogen peroxide into water and molecular oxygen in two steps (Aldiniet al., 2011; Gomeset al., 2012). Decreased catalase activity increases oxidative stress and perpetuates inflammatory responses (Rogalaet al., 2015). This phenomenon has been confirmed in the serum of patients with asthma (M. Sahiner et al., 2018; Ammar, 2022). Glutathione peroxidase regenerates hydrogen peroxide and lipid hydroperoxides into water and alcohol. During this process, the glutathione of this enzyme is oxidized to glutathione disulfide and is subsequently restored by the enzyme glutathione reductase. This enzymatic process is crucial in neutralizing oxygen species (Brown et al., 2007).

The pathophysiological changes in asthma are closely associated with the increased production of ROS, which are released due to the cascade of inflammatory mediators. Conversely, a decrease in antioxidant levels exacerbates the inflammatory responses in asthma patients. Inflammatory cells such as activated eosinophils, neutrophils, monocytes, and macrophages, as well as stationary cells like epithelial cells and smooth muscles, can generate ROS (M. Sahiner et al., 2018). These oxidants contribute to airway smooth muscle contraction, increased lung vascular permeability, airway epithelial damage, heightened mucus secretion, bronchial hyperreactivity, and ultimately the severity of asthma (Kozan et al., 2016; M. Sahiner et al., 2018).

Our histopathological findings revealed the accumulation of mucus, inflammation, and muscle hypertrophy in the sensitized group, aligning with prior research (M. Ezz-Eldina et al., 2020). These pathological results indicate that the structural changes in the respiratory tracts of asthmatic rats are concurrent with inflammation and alterations in oxidative stress factors, consistent with earlier studies (Kozan et al., 2016; M. Sahiner et al., 2018). In our study, the treatment groups that received a high concentration of the plant extract and those treated with RA exhibited a significant reduction in lung inflammation, muscle hypertrophy, and mucus plaques compared to the sensitized group. Previous studies have highlighted the protective effects of Myrtenol in reducing inflammation and the severity of asthma by increasing antioxidant activity and reducing oxidant levels in allergic asthma in rats (Rajizadeh et al., 2019). The hydroalcoholic extract of *M. officinalis* has been reported to exhibit anti-inflammatory effects in a carrageenan-induced foot edema model, further confirming its antioxidant properties (Draginic et al., 2022). It has been reported that Plantago primary hydroalcoholic extract in asthmatic rats induced by citric acid significantly reduced the mean number of mast cells and alveolar sac hemorrhage, as well as decreased the thickness of alveolar epithelium (Farokhi and Khaneshi, 2013). Administration of medicinal herbs Brassica napus L. or a polyherbal formulation on OVA-induced asthma ameliorated lung histopathological changes, including reduced eosinophil numbers, goblet cell hyperplasia, and smooth muscle layer thickness, in comparison to the sensitized group (Hamzeloo-Moghadam et al., 2022; Neamati et al., 2013).

Dexamethasone is commonly used as a standard drug to alleviate the severity of asthma attacks, attributed to its ability to reduce inflammation and oxidative stress activity. Various studies have supported this assertion (Eftekhar et al., 2018; Salehi et al., 2011a; M. Ezz-Eldina et al., 2020). In this study, the group treated with dexamethasone exhibited significant improvements in oxidative stress factors, inflammatory cell count, and lung pathology. These improvements were accompanied by a reduction in inflammatory cells and oxidants, an increase in antioxidant activity, and an overall improvement in lung tissue. Pathological samples showed reduced mucus secretion, decreased lung inflammation, and improved muscle hypertrophy. The effects of the plant extract were comparable to dexamethasone in reducing inflammatory cells and oxidants, increasing antioxidant activity, and improving lung tissue. The plant extract demonstrated even greater efficacy in some factors than dexamethasone. Other studies investigating the antipyretic effects of the hydroalcoholic extract of Ocimum basilicum leaves or Myrtenol in a rat model of ovalbumin-induced asthma also observed reductions in lung pathology (Eftekhar et al, 2018; Rajizadeh et al., 2019).

Similarly, treatment with RA (Costa et al., 2012) in a mouse model of respiratory allergy caused by the mite Blomia tropicalis resulted in reduced mucus secretion and decreased inflammatory cell presence around the bronchi, consistent with the findings of this study. The hydroalcoholic extract of *M. officinalis* and RA likely exert their anti-inflammatory and antioxidant effects by inhibiting cyclooxygenase enzymes, reducing inflammatory cytokines (Vaez et al., 2011), reducing histamine levels by RA (Oh et al., 2011), and the presence of various antioxidants. These effects were observed in rats sensitized by ovalbumin in this study.

The biochemical and histopathological findings in the present study show that the hydroalcoholic extract of *M. officinalis* and its active ingredient, RA, can effectively limit inflammatory cells, suppress airway inflammation and mucus production, and smooth muscle cell hypertrophy in bronchial asthma. The plant extract and RA could also improve oxidative stress by reducing No_2_ and MDA levels and improving thiol, SOD, and CAT activities. If future clinical studies confirm this effect, it can be used as a natural medicine that effectively reduces asthma symptoms.

## Conflicts of interest

The authors have declared that there is no conflict of interest.
